# Anti-Stokes Fluorescent Probe with Incoherent Excitation

**DOI:** 10.1038/srep04059

**Published:** 2014-02-12

**Authors:** Yang Li, Shifeng Zhou, Guoping Dong, Mingying Peng, Lothar Wondraczek, Jianrong Qiu

**Affiliations:** 1 State Key Laboratory of Luminescent Materials and Devices, Institute of Optical Communication Materials, School of Materials Science and Technology, South China University of Technology, Guangzhou 510640, China; 2 Otto Schott Institute, University of Jena, Jena07743, Germany

## Abstract

Although inorganic anti-Stokes fluorescent probes have long been developed, the operational mode of today's most advanced examples still involves the harsh requirement of coherent laser excitation, which often yields unexpected light disturbance or even photon-induced deterioration during optical imaging. Here, we demonstrate an efficient anti-Stokes fluorescent probe with incoherent excitation. We show that the probe can be operated under light-emitting diode excitation and provides tunable anti-Stokes energy shift and decay kinetics, which allow for rapid and deep tissue imaging over a very large area with negligible photodestruction. Charging of the probe can be achieved by either X-rays or ultraviolet-visible light irradiation, which enables multiplexed detection and function integration with standard X-ray medical imaging devices.

The exploration and assessment of structural and functional processes in cells, tissue and other complex systems requires fluorescence optical imaging as a tool for rapid and non-invasive biological monitoring with high spatial and temporal resolution^1^,^2^,^3^. The versatility of today's imaging devices is largely dictated by the photo-physical and photochemical properties of photonic probes. Current methods of optical imaging which employ genetically encoded proteins, organic dyes, semiconductors or persistent phosphors usually exhibit temporal variations in their fluorescence due to their inherently weak photo-stability, blinking behavior or persistent emission characteristics, thus posing great limitations in tracking biological processes^4^,^5^,^6^,^7^. Alternative photonic probes based on anti-Stokes emission from inorganic phosphor materials appear not only highly suitable as a possible solution for these issues, but they also provide new functionality such as nano-scale thermometry and delayed detection^8^,^9^,^10^,^11^. Despite their apparent advantages, however, such anti-Stokes probes rely strictly on higher-order excitation. This typically requires high-intensity illumination with laser sources, which often leads to photon-induced deterioration of the analyte. A significant improvement of the anti-Stokes emission efficiency of the employed materials may potentially decrease the operation threshold of the probe, but the fundamental limitation of coherent excitation is still present^12^,^13^.

Here, we propose and experimentally demonstrate an effective anti-Stokes photonic probe which operates under incoherent excitation. The probe is engineered to such a way as being capable of separately confining high density electrons and holes for a rather long duration and facilitating their recombination through desired emitters. As shown in Figure 1, upon charging (electron beam, UV light, or X rays), electron-hole pairs are generated and the excited electrons are firmly captured on purposefully introduced trapping states. The mass out-migration of electrons from the ground band leads to the formation of a “satellite valence band” in a high-energy position. Optical pumping with a low- energy incoherent light source at a wavelength of *λ*_1_ is then sufficient to extract electrons from this reservoir, followed by electron-hole recombination through an incorporated emitter which is accompanied by the emission of a photon with lower wavelength, *λ*_2_. The key feature of this scheme is that the location of the introduced band and the energy gap of the emitter can be rationally tuned. As a result, anti-Stokes emission under incoherent excitation can be obtained, with the added benefits of continuously adjustable frequency shift and broadband excitation. In addition to the anticipated advantage of strongly reduced photo-bleaching during imaging, the approach also overcomes the stringent constraints of the inherently small beam diameter of lasers, thus offering the possibility of rapid optical imaging of tissues over a much larger area^14^,^15^.

## Results

### Establishment of an anti-Stokes photonic probe

The establishment of an anti-Stokes photonic probe requires a material system which is suitable for building a mid-gap electron reservoir. To this end, we have pursued the mixed oxide system Zn-Ga-Ge-O, which has been proven capable of supporting high defect densities, thought to be associated primarily with Ge^4+^ vacancies (V_Ge_) and O^2−^ vacancies (V_O_), as well as Zn deficiency^16^. As the optimal emitter, Cr^3+^ was chosen and introduced exemplarily into Zn_3_Ga_2_Ge_2_O_10_ as a probe material. Making use of its defect capacity, such Zn_3_Ga_2_Ge_2_O_10_:Cr^3+^has been demonstrated as a near-infrared photo-emitter with surprisingly persistent afterglow^16^. Here, we target the operating waveband in the biological transparency window of 700–1100 nm. Upon optical charging of the probe, a prominent electron spin resonance (ESR) appears at a *g*-value of 1.9996 (3374.9 G, Figure 2b). This signal is readily assigned to the presence of trapped electrons^17^. We also observe that the ESR signal and, hence, the defect state persists over an extended period of time, i.e., >6 hrs (Figure 2b), what is a clear confirmation of the optical storage function of the material. The slight decrease in signal intensity with holding time is attributed to quantum tunneling^18^. In the photocurrent excitation (PCE) spectra obtained at various temperatures between 20 and 300 K (Figure 2c), a broad excitation band which covers the spectral range of about 250–450 nm is observed. This firmly supports the feasibility of energy charging by ultraviolet-visible (UV-vis) light excitation. Thus, as a preliminary conclusion and confirmation of previous studies, an electron reservoir can effectively be introduced into this specific material system.

**Figure 1:**
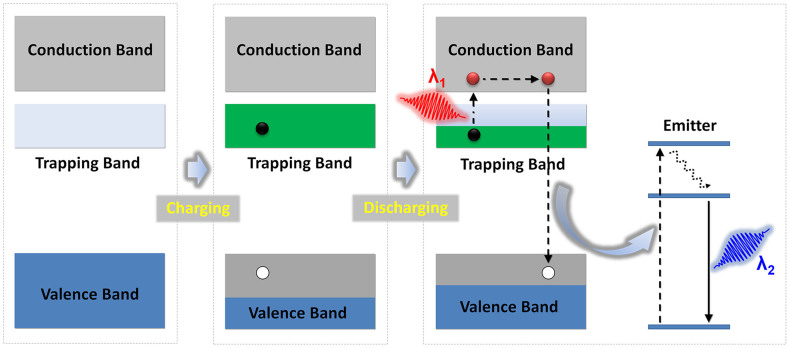
Energy band model depicting anti-Stokes emission under incoherent excitation. During an optical charging process, the gap between valence and conduction band is decreased by generating an electron-hole pair. The trapped electron mitigates into a satellite trapping band, from where it can be extracted by optical pumping. Emission of a secondary photon with lower wavelength than occurs through recombination of the electron-hole pair.

**Figure 2:**
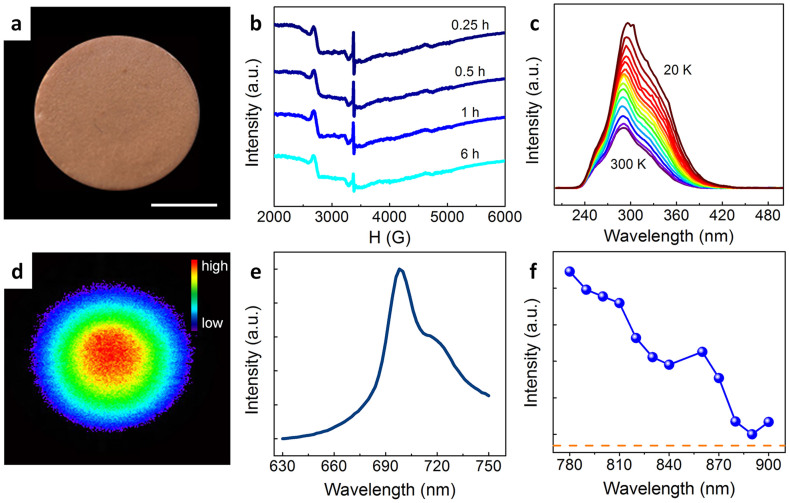
Properties of an exemplary sample for anti-stokes non-coherent fluorescence probing. (a) Photograph of an as-prepared disc sample. Scale bar indicate a size of 4 mm. (b) Time-dependent electron spin resonance spectra of the sample after charging. (c) Temperature-dependent photocurrent excitation spectra for the range of 20 ~ 300 K. (d) Luminescence image taken at the interval of 24 hours after charging of the sample for illumination with an incoherent LED operating at a wavelength of 800 nm (here: 7.87 mW/cm^2^). (e) Static photoluminescence spectrum under excitation at 800 nm and (f) the corresponding photoluminescence excitation spectra monitored at 700 nm of the charged sample. Before measurements, effective optical charging of the probe can be done by UV-vis light for 5 min, the measurements were taken at the interval of 24 hours after charging.

The photoluminescence (PL) spectrum of the charged sample features a broad emission band which is located at ~700 nm (for incoherent excitation at 800 nm with a light emitting diode (LED), Figure 2e). This emission band can be conclusively assigned to the ^2^E → ^4^A_2_ transition of octahedrally coordinated trivalent chromium, ^VI^Cr^3+^ 19. Of importance for the application of the material as fluorescent probe, the emission band can be readily observed even for a fairly low density of excitation irradiation (here: 7.87 mW/cm^2^, Figure 2d) and plenty of electrons are still detained in the traps even at 24 h after charging. This underlines the potential of the material for low-threshold incoherently excited anti-Stokes emission probing. Noteworthy, no similar luminescence phenomenon can be observed even under high-density coherent laser excitation for the uncharged sample, what confirms the scheme shown in Figure 1 as the basis for the observed photoemission behavior. Figure 2f shows the photoluminescence excitation spectrum of the charged sample monitored at 700 nm. The effective PLE band covers the spectral region from 780 to 900 nm, within which the ^VI^Cr^3+^ ion does normally not show any absorption. That is, luminescence occurs as a result of energy transfer from the trapped defect states (Figure 1) to the ^VI^Cr^3+^ emitter. The broad width of the excitation band provides a possibility for adjusting the wavelength of pumping source.

### Transition kinetics of the emitters

**Figure 3:**
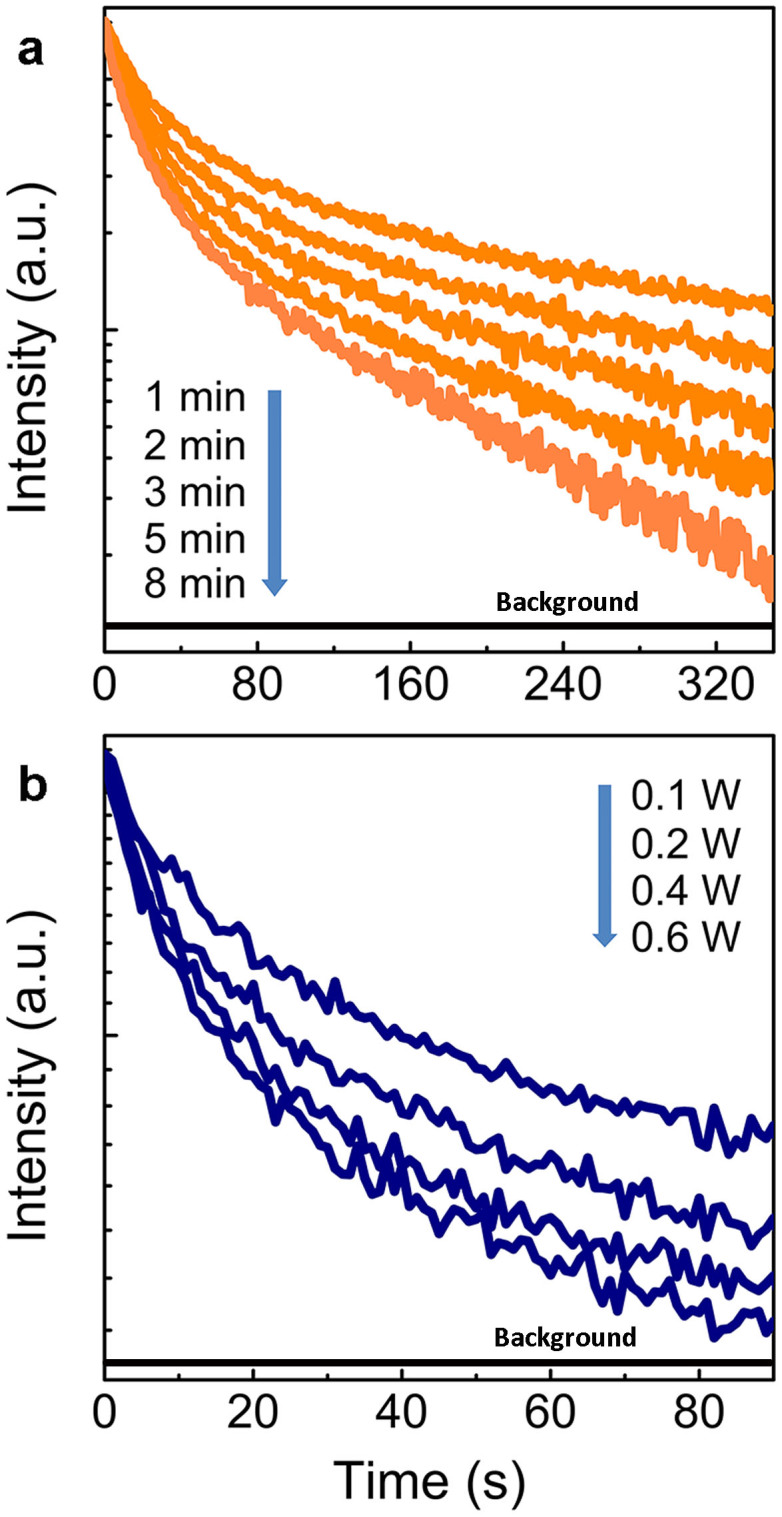
Anti-Stokes luminescence kinetics. Photoluminescence decay in dependence on duration of excitation (a. constant excitation intensity 0.03 W) and excitation intensity (b. constant excitation duration 2 min) for excitation with a 980 nm laser diode (LD) and monitoring the emission intensity at a wavelength of 700 nm. Before measurements, effective optical charging of the probe can be done by UV-vis light for 5 min, the measurements were taken at the interval of 24 hours after charging.

Another intriguing property of the anti-Stokes luminescence based on this energy storage mechanism is the possibility of engineering the radiative transition kinetics of the emitter. In Figure 1, we propose that photo-excited electrons would be captured in traps. This would yield a first fast, subsequently gentle and continuous relaxation process, accompanied by a sustained release of energy. To validate this concept, we consider the luminescence decay kinetics of the sample. The decay profiles are presented in Figure 3. The decay curves don't exhibit a typical single exponential shape (Figure s1). For the initial consideration, the surprisingly slow second decay step is the most relevant. It stands in great contrast to the relatively short lifetime of a few milliseconds which is typically found in rare-earth doped fluorites with maximum anti-Stokes emission performance^20^. Here, we observe, in comparison, an increase by a factor of ~10^3^ in the timescale of decay, i.e., seconds rather than milliseconds. This makes the present material an almost ideal candidate for application as a robust probe with delayed detection capability (It has been proposed that late time-gating could be used to image molecular or quantum dot imaging probes in the presence of this interference. It is claimed that emission lifetime of nanoparticles larger than several microseconds or milliseconds are considered to be sufficiently long to permit late time-gated imaging.)^21^. It is also observed that the decay rate exhibits a strong positive dependence on excitation time and excitation intensity: it decreases with longer pumping duration and increases with decreasing excitation intensity. We relate this previously not reported phenomenon to the specific nature of the optical energy storage mechanism.

### Stability and reproducibility

**Figure 4:**
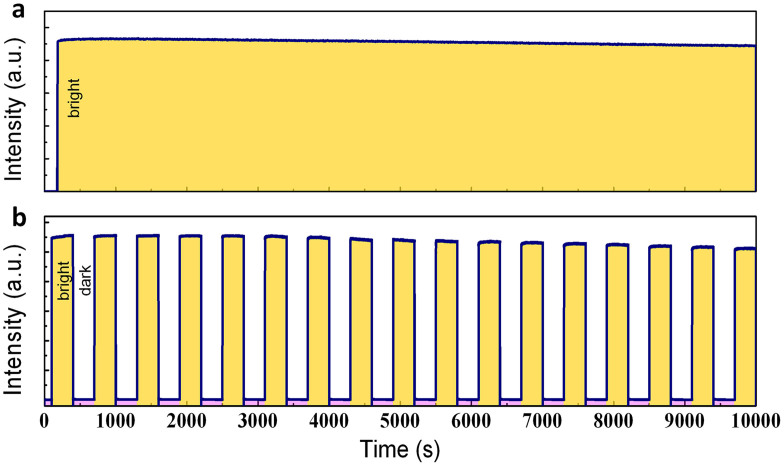
Anti-Stokes luminescence stability and reproducibility. (a) Evolution of luminescence monitored at 700 nm under continuous illumination with an incoherent LED (7.87 mW/cm^2^) operating at a wavelength of 800 nm. (b) Reproducibility of anti-Stokes luminescence generation during 17 on/off cycles over a period of 10000 s. Before measurements, effective optical charging of the probe can be done by UV-vis light for 5 min, the measurements were taken at the interval of 24 hours after charging.

The stability and reproducibility of the anti-Stokes luminescence behavior of Zn_3_Ga_2_Ge_2_O_10_:Cr^3+^ are illustrated in Figure 4. Even after several hours of continuous irradiation with a LED (7.87 mW/cm^2^) operating at a wavelength of 800 nm, the anti-Stokes emission remains essentially unaltered in intensity (I_background_/I_fluorescence_ ≈ 1/1000, Figure 4a). A similar result was obtained for cyclic operation over 17 individual on/off cycles of 10 min, where a maximum signal degradation of 8% was observed (Figure 4b). Both results indicate the high photo-stability of the material.

## Discussion

**Figure 5:**
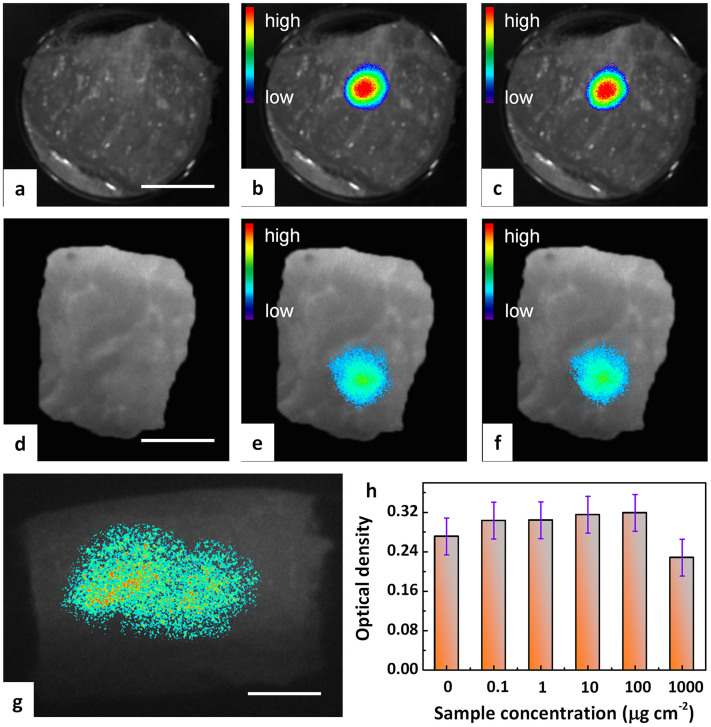
*A*nti-Stokes fluorescence tissues imaging of pork tissue with incoherent excitation. (a–c and g) show the application of *ex situ* optical charging, (d–f) represent X-ray *in situ* charging: (a) Pre-injection autofluorescence image. (b) 60 min post-injection fluorescence image and (c) representative reproduction of (b) after 1 on/off cycling. A 980 nm laser diode was employed as the excitation source and the monitoring wavelength was set at ~700 nm. (d) Post-injection autofluorescence image without charging. (e) Post-injection fluorescence image after external X-ray charging and (f) representative reproduction of (e) after 1 on/off cycling. (g) Deep and large-area fluorescence imaging of pork tissue for an injection depth of 1 cm. A 940 nm LED was employed as the excitation source for imaging and the monitoring wavelength was set at ~700 nm. Scale bars are 15 mm for panels a–g. (h) *In-vitro* viability of BMSCs (bone mesenchymal stem cells) incubated with particulate Zn_3_Ga_2_Ge_2_O_10_:0.5Cr^3+^ as anti-Stokes probe at different concentrations for 3 days. Each data point represents the mean value of at least three independent experiments.

The experimental demonstration of anti-Stokes luminescence imaging with incoherent excitation was performed by testing the visibility of the probe inside biological tissue. For that, fluorescence imaging was done by direct injection of disperse particles of Zn_3_Ga_2_Ge_2_O_10_:0.5Cr^3+^ into pork tissue. Charging was performed *ex situ* as well as *in situ*. Typical fluorescence micrographs are summarized in Figure 5. Figures 5 b–c represent exemplary images taken at the same sample location within a time window of 100 min for *ex situ* optical charging (Xenon short-arc lamp) of the probe. They clearly show the presence of the probe and its high optical stability which we find, within the observation timescale, in the range of the sensitivity of detection. External (*in situ*) X-ray activated and recharging of the probe is demonstrated in Figures 5d–f. The probe signal can also be clearly be distinguished from the autofluorescence of the tissue. This shows that the probe can be exploited for multiplexed excitation and detection. The combination of *in situ* and *ex situ* X-ray charging capability enables integration with established X-ray medical imaging techniques such as radiography and computed tomography.

The application of an incoherent excitation source potentially enables a notable increase of detection area, as large-area excitation schemes can be employed with less difficulty, for example using large aperture LED sources instead of focused laser beams. While the latter typically enable a detection area ~ 0.5 cm × 0.5 cm, in Figure 5 g, we test simultaneous excitation of an area of ~6 cm^2^. Clearly, luminescent read-out is achieved over the largest part of excitation area. When the excitation and detection wavelengths locate at near-infrared biological window, the luminescent image can still be visualized even in the case of a large injection depth of 1 cm.

In conclusion, we discuss incoherent anti-Stokes luminescent probing of biological tissue with Zn_3_Ga_2_Ge_2_O_10_:0.5Cr^3+^. We show that the high defect capacity of this material enables effective optical charging, before (*ex situ*) or after (*in situ*) injection into the analyte. We demonstrate incoherent activation for large-area (~6 cm^2^) as well as large-depth (~1 cm) detection capability. By regulating the energy level position of the electron reservoir and excitation parameters such as energy, intensity and duration, it provides tunable decay kinetics. Charging of the probe can be done by either X-rays or UV-vis light, what enables multiplexed detection and function integration with standard X-ray medical imaging devices. We believe that overcoming the stringent requirement of coherent excitation for achieving anti-Stokes emission opens new paths for advanced photon management^13^,^22^,^23^.

## Methods

### Sample synthesis

Different synthesis methods were employed to fabricate samples with various particle sizes. To prepare micro-sized powders, the solid state reaction method was used. The synthesis procedure of Zn_3_Ga_2_Ge_2_O_10_:0.5Cr^3+^ as an exemplary material candidate for incoherent anti-Stokes luminescent probing of soft tissue was adopted from Ref. 16 Sample powders and compacted pellets were produced through a conventional solid state reaction of stoichiometric batches of GeO_2_, ZnO, Ga_2_O_3_ and Cr_2_O_3_ (≥4N). The reaction comprised a three-step thermal treatment, i.e., initial calcination at 1000°C for 4 h, secondary calcination at 1150°C for 6 h. A part of the obtained powder (~2 g) was pressed into disc-shaped samples with diameters of ~10 mm using an uniaxial hydraulic press, and finally sintered at 1350°C for 30 min. To prepare nano-sized powders, a sol-gel procedure was employed. For that, an aqueous precursor solution was prepared by dissolving stoichiometric amounts of zincacetate (Zn(CH_3_COO)_2_, 99%), galliumnitrate (Ga(NO_3_)_3_, 99.99%), tetraethyl-germanate (C_8_H_20_Ge, 99.999%), and chromiumnitrate (Cr(NO_3_)_3_, 99%). The solution was stirred vigorously at room temperature about 1 h until it became limpid. During the stirring process, citric acid was added into the solution to form chelate complexes. The solution was then heated to 70°C until a gel was obtained. The wet gel was dried at 110°C for 24 h. The dry gel was finally calcinated at 900°C for 3 hrs. In this way, particles with a size of about 50 nm ~ 300 nm were obtained (Figure s3–s4).

### Material characterization

The as-obtained material was analyzed by X-ray diffraction (Cu/Kα), confirming the presence of Zn_3_Ga_2_Ge_2_O_10_ as the sole crystalline phase. In this lattice, Cr^3+^ was supposed to precipitate on the octahedral sites of Ga^3+^ 24. To study the presence of paramagnetic defects, ESR spectra were recorded with an X-band spectrometer (Bruker A300). Before measurements, effective optical charging of the probe can be done by UV-vis light for 5 min. PCE spectra were measured in a multi-channel spectrometer under monochromatic excitation obtained by the combination of a 300 W Xenon lamp and a monochromator. Before measurement, two gold electrodes with a separation distance of ~1 mm were deposited on the sample for masking. The photocurrent was measured with a digital electrometer under a DC voltage of ~200 V. Spectra were recorded over the temperature range of 20–300 K, employing a closed-cycle liquid He cryostate. Room-temperature PL, PLE spectra and decay curves were measured with a high-resolution spectrofluorometer (Edinburgh Instruments FLS920) equipped with a 500 W Xenon lamp, a 800 nm LED and a 980 nm LD as excitation sources (980 nm LD and 800 nm LED were chosen to demonstrate both the effect of wavelength and intensity of the excitation light on decay rates). Energetic charging of the sample is achieved by exposure to an external field. Effective charging of the probe can be done by UV-vis light/X-ray for 5 min/20 min; the measurements were taken at the interval of 24 hours after charging. The absolute photoluminescence quantum yields (*QY*) of the micro-sized and nano-sized powders were determined on an FLS920 spectrometer (Edinburgh, UK) with an integration sphere attachment. The QY (pre-irradiated by a UV light) under the excitation of 810 nm light of micrometer size powders is 1.5% (Figure s2), comparable to those of NIR emitting quantum dots and carbon nanotubes, but the QY of nano-size powders is less than 1%. Here, we employed either X-rays (Philips X-pert pro M, 15 min) or UV-vis light (xenon arc lamp, 5 min) and verified the charging process by ESR.

### Incoherent anti-Stokes fluorescent probing

As a proof of concept, Zn_3_Ga_2_Ge_2_O_10_:Cr^3+^ was employed as an anti-Stokes luminescent probe with incoherent excitation for imaging pork tissue. Nano-particles (Zn_3_Ga_2_Ge_2_O_10_:Cr^3+^, dispersed in ordinary saline (100 mg/ml)) were injected into tissue at various injection depths (0.1–1 cm). *Tissues* imaging was performed with a modified ZKKS-MI-III (Zhongke Kaisheng, Medical Technology Co.,Ltd., Guangzhou, China) *tissues* imaging system using an external 980 nm LD, 800 nm LED and 940 nm LED as the excited source and an Andor EMCCD as the signal collector. Super-cold filters (Asahi, ZSC1100), long-pass filters (Asahi, ZVL630), and band-pass filters (Asahi, ZBPB147) were inserted to block the excitation light and transmit the emission light. The images were analyzed with Zhongke Kaisheng Imaging Software. In a typical experiment of imaging with *ex situ* optical charging, the charged particles were injected intramuscularly into the pork tissue. In a typical experiment of imaging with *in situ* X-ray charging, the uncharged particles were injected intramuscularly into the pork tissue and externally charged by using X-rays. An incoherent 940 nm LED with large aperture (7.87 mW/cm^2^) was employed as the excitation source for imaging. Deep-tissue imaging was tested by injecting the probe into various depths from 0.1 to 1 cm.

### Cytotoxicity

In the cytotoxicity experiment, BMSCs (bone mesenchymal stem cells) were cultured in HDMEM medium (Invitrogen, USA) containing 10% heat-inactivated FBS (fetal bovine serum) at 37°C in the humidified atmosphere with 5% CO_2_. The cells were seeded in 96-well plates at a density of 10 × 10^3^ cells/cm^2^ and grew overnight prior to studies. Then, the cells were incubated with fresh media with gradient doses of anti-Stokes fluorescent probes (from 0.1 μg/cm^2^ to 1000 μg/cm^2^). After incubation for 3 days, 20 μL of MTT (thiazolyl blue tetrazolium bromide, 10 mg/mL, Sigma–Aldrich, USA) solution was added to each well of the plate, and then the plate was incubated at 37°C for 4 hrs. Finally, the cells were lysed using DMSO (Sigma, USA). A microplate reader (Bio-Rad 680, USA) was applied to monitor the absorbance of the supernatants at 495 nm. This experiment was repeated for three times. We observed cellular viabilities of greater than 95% in the presence of injections with a concentration of ≤100 μg/cm^2^ (Figure 5h), indicating relatively low toxicity of the employed probe.

## Author Contributions

J.R.Q. proposed the idea and designed the experiments. Y.L. prepared the samples. Y.L., S.F.Z., G.P.D. and M.Y.P. were responsible for luminescence and J.R.Q., S.F.Z. analyses and data reproduction. S.F.Z., J.R.Q., Y.L. and L.W. evaluated the data. S.F.Z. and Y.L. wrote the manuscript and J.R.Q. and L.W. checked the manuscript. All authors were involved in the discussion of the experimental results.

## Supplementary Material

Supplementary Information Supporting information

## References

[b1] Ozeki Y. *et al.* High-speed molecular spectral imaging of tissue with stimulated Raman scattering. Nat. Photon. 6, 845–851 (2012).

[b2] Saito K. *et al.* Luminescent proteins for high-speed single-cell and whole-body imaging. Nat. Commun. 3, 1262(1)–1262(9) (2012). 23232392 10.1038/ncomms2248 PMC3535334

[b3] Nicholas G. *et al.* In vivo three-photon microscopy of subcortical structures within an intact brain. Nat. Photon. 7, 205–209 (2013). 10.1038/nphoton.2012.336 PMC3864872 24353743

[b4] Bouzigues C., Gacoin T. & Alexandrou A. Biological applications of rare-earth based nanoparticles. ACS Nano. 5, 8488–8505 (2011). 21981700 10.1021/nn202378b

[b5] Michalet X. *et al.* Quantum dots for live cells, in Vivo imaging, and diagnostics. Science 307, 538–544 (2005). 15681376 10.1126/science.1104274 PMC1201471

[b6] Chermmont D. Q. L. *et al.* Nanoprobes with near-infrared persistent luminescence for *in vivo* imaging. Proc. Natl. Acad. Sci. U.S.A. 104, 9266–9271 (2007). 17517614 10.1073/pnas.0702427104 PMC1890483

[b7] Zenobi R. & Deckert V. Scanning near-field optical microscopy and spectroscopy as a tool for chemical analysis. Angew. Chem. Int. Ed. 39, 1746–1756 (2000). 10.1002/(sici)1521-3773(20000515)39:10<1746::aid-anie1746>3.0.co;2-q 10934352

[b8] Liu F. *et al.* Photostimulated near-infrared persistent luminescence as a new optical read-out from Cr^3+^-doped LiGa_5_O_8_. Sci. Rep. 3, 1554; 10.1038/srep01554 (2013). 23532003 10.1038/srep01554 PMC3609016

[b9] Zhou J., Liu Z. & Li F. Upconversion nanophosphors for small-animal imaging. Chem. Soc. Rev. 41, 1323–1349 (2012). 22008740 10.1039/c1cs15187h

[b10] Gorris H. H. & Wolfbeis O. S. Photon-upconverting nanoparticles for optical encoding and multiplexing of cells, biomolecules, and microspheres. Angew. Chem. Int. Ed. 52, 3584–3600 (2013). 10.1002/anie.201208196 23450698

[b11] Zou W., Visser C., Maduro J. A., Pshenichnikov M. S. & Hummelen J. C. Broadband dye-sensitized upconversion of near-infrared light. Nat. Photon. 6, 560–564 (2012).

[b12] Fischer L. H., Harms G. S. & Wolfbeis O. S. Upconverting nanoparticles for nanoscale thermometry. Angew. Chem. Int. Ed. 52, 4546–4551 (2011). 10.1002/anie.201006835 21495125

[b13] Wang F. *et al.* Tuning upconversion through energy migration in core-shell nanoparticles. Nat. Mater. 10, 968–973 (2011). 22019945 10.1038/nmat3149

[b14] George B. *et al.* Valency conversion of samarium ions under high dose synchrotron generated X-ray radiation. Phy. Status Solidi C 8, 2822–2825 (2011).

[b15] Heike E. H. *et al.* Radiation dosimetry using optically stimulated luminescence in fluoride phosphate optical fibres. Opt. Mat. Express. 2, 1648–1656 (2012).

[b16] Pan Z., Lu Y. & Liu F. Sunlight-activated long-persistent luminescence in the near-infrared from Cr^3+^-doped zinc gallogermanates. Nat. Mater. 11, 58–63 (2012). 22101812 10.1038/nmat3173

[b17] Schreurs J. w. H. Study of some trapped hole centers in X-irradiated alkali silicate glasses. J. Chem. Phys. 47, 818–830 (1967).

[b18] Avouris P. & Morgan T. N. A tunneling model for the decay of luminescence in inorganic phosphors: The case of Zn_2_SiO_4_:Mn. J. Chem. Phys. 74, 4347–4355 (1981).

[b19] Kück S. Laser-related spectroscopy of ion-doped crystals for tunable solid-state lasers. Appl. Phys. B 72, 515–562 (2001).

[b20] Balda R., Garcia-Adeva A. J., Voda M. & Fernández J. Upconversion processes in Er^3+^-doped KPb_2_Cl_5_. Phys. Rev. B 69, 205203(1)–205203(8) (2004).

[b21] Luo G. *et al.* In vivo time-gated fluorescence imaging with biodegradable luminescent porous silicon nanoparticles. Nat. Commun. 4, 2326(1)–2326(7) (2013). 23933660 10.1038/ncomms3326 PMC4154512

[b22] Wang H., Batentschuk M., Osvet A., Pinna L. & Brabec C. J. Rare-earth ion doped up-conversion materials for photovoltaic applications. Adv. Mater. 23, 2675–2680 (2011). 21823249 10.1002/adma.201100511

[b23] Huang X., Han S., Huang W. & Liu X. Enhancing solar cell efficiency: the search for luminescent materials as spectral converters. Chem. Soc. Rev. 42, 173–201 (2013). 23072924 10.1039/c2cs35288e

[b24] Mathieu A. *et al.* Considerable improvement of long-persistent luminescence in germanium and tin substituted ZnGa_2_O_4_. Chem. Mat. 25, 1600–1606 (2013).

