# Supplementary material for: Anti-Stokes Fluorescent Probe with Incoherent Excitation
Source: Sci Rep. 2014 Feb 12;4:4059. doi: 10.1038/srep04059 (PMC3921629; doi:10.1038/srep04059)
Supplement: Supplementary Information — Supporting information [file srep04059-s1.doc]

*Supporting information*

**Anti-Stokes Fluorescent Probe with Incoherent Excitation**

Yang Li^1^, Shifeng Zhou^1^,*, Guoping Dong^1^, Mingying Peng^1^, Lothar Wondraczek^2^ and Jianrong Qiu^1^,*

^1^*State Key Laboratory of Luminescent Materials and Devices, Institute of Optical Communication Materials, School of Materials Science and Technology, South China University of Technology, Guangzhou 510640, China*

*^2^ Otto Schott Institute, University of Jena, Jena 07743, Germany*

**Measurement of Absolute Quantum Yield.**

The absolute photoluminescence quantum yields (*QY*) of the micro-sized and nano-sized powders at room temperature were determined on an FLS920 spectrometer (Edinburgh, UK) with an integration sphere attachment. A 10 mm path length quartz cuvette for the solution sample is set in the integrating sphere. The absolute photoluminescence QY is given by:

$$\text{QY=}\frac{\int L_{Sample}}{\int E_{\text{R}eference}-\int E_{Sample}}$$

*L_Sample_* is the photoluminescence intensity of the sample; *E_Reference_* and *E_Sample_* are the excitation light intensities of the reference and the sample, respectively. The pure solvent (ultrapure water) was employed as reference.

Figure Legend

Figure s1 Excitation duration dependent photoluminescence decay curves (excitation power: 0.03 W, wavelength: 980 nm) and the fitted curves. The emission intensity at 700 nm was monitored.

Figure s2 Absolute photoluminescence quantum yield (QY) of the micrometer size powders (QY=1.5%). The excitation wavelength is 810 nm, and the emission band is from 650 nm to 800 nm.

Figure s3 SEM image of the nano-sized powders.

Figure s4 Dynamic light scattering spectrum of the nano-sized powders


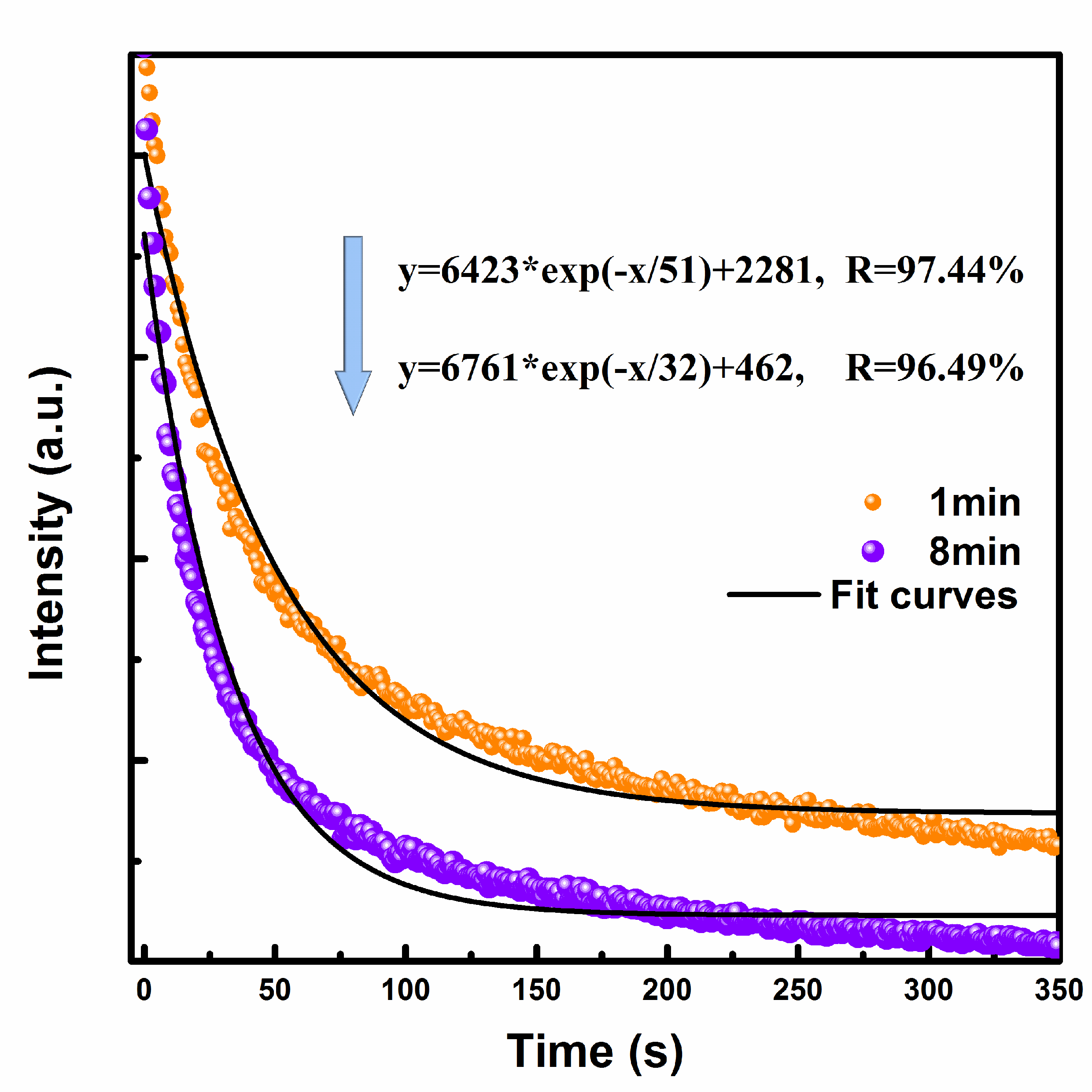


Figure s1


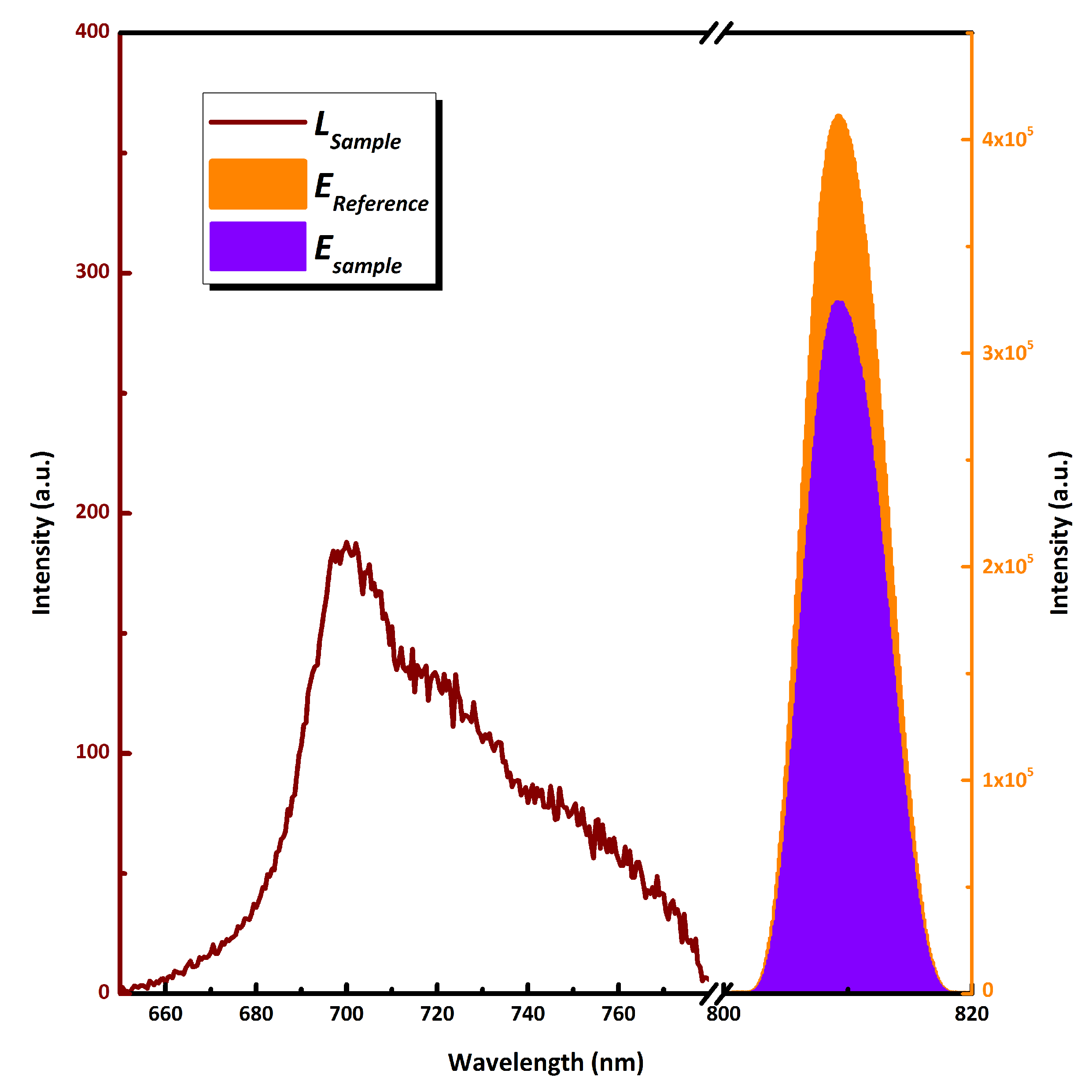


Figure s2


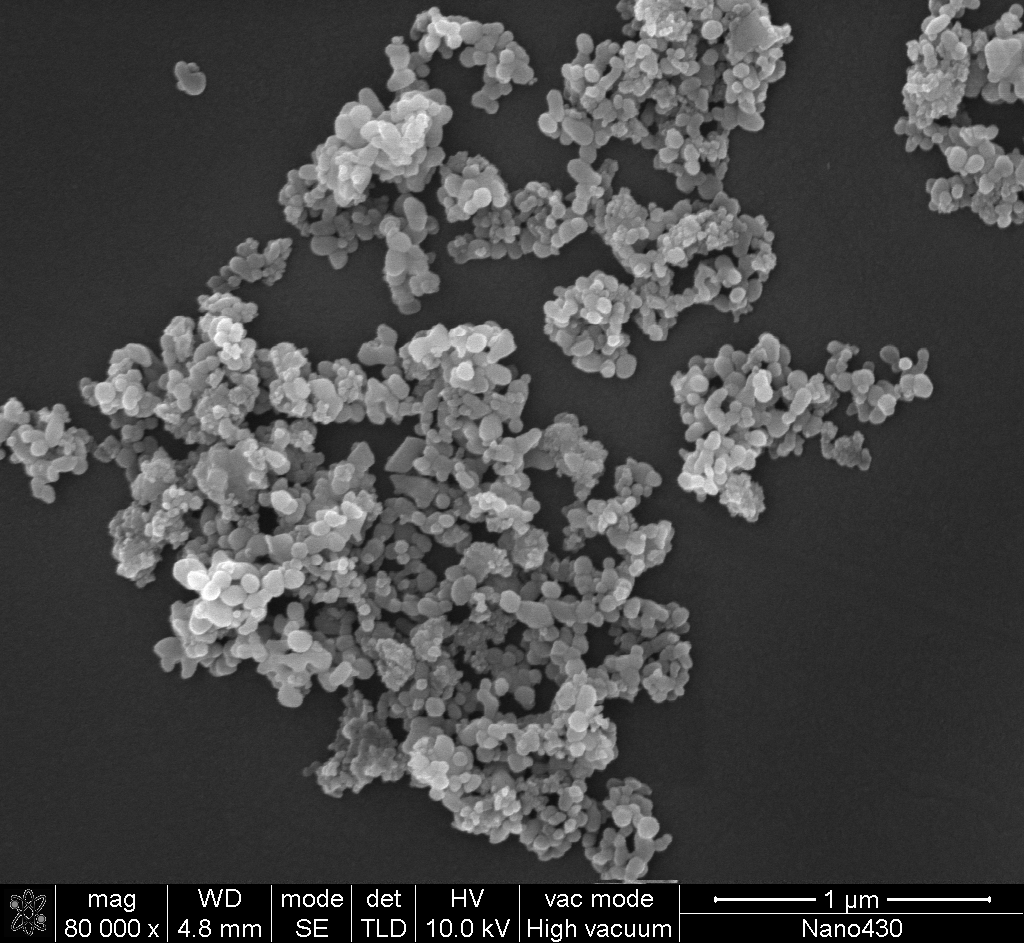


Figure s3


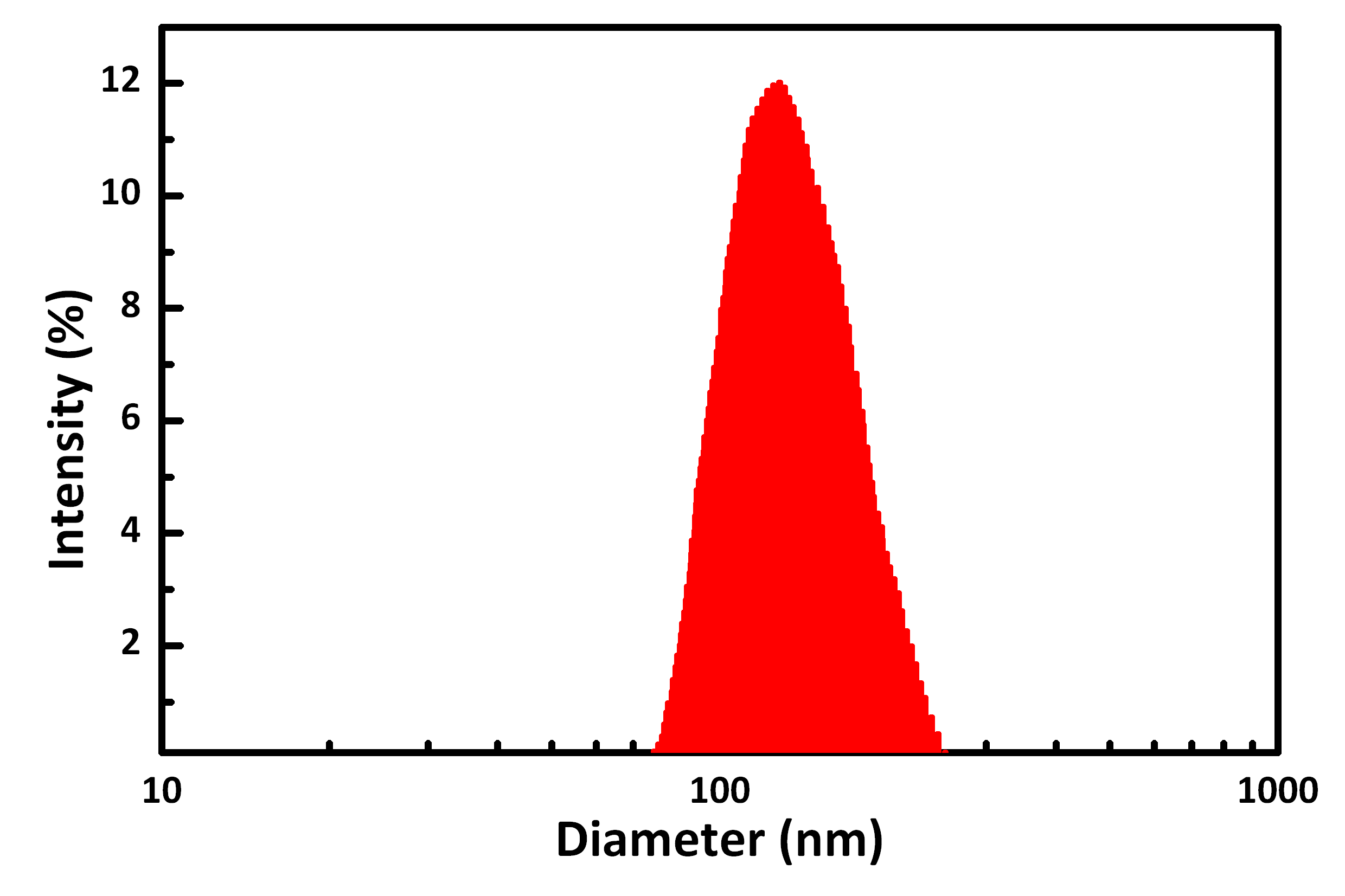


Figure s4
